# The cloud paradigm applied to e-Health

**DOI:** 10.1186/1472-6947-13-35

**Published:** 2013-03-14

**Authors:** Jordi Vilaplana, Francesc Solsona, Abella, Rosa Filgueira, Josep Rius

**Affiliations:** 1 Computer Science Department, University of Lleida, Jaume II 69, Lleida, 25001, Spain; 2 Unitat de Tabaquisme of Hospital Santa Maria de Lleida, , Alcalde Rovira Roure, 44, Lleida, 25198, Spain; 3 Edinburgh Data-Intensive Research Group, School of Informatics, The University of Edinburgh, Edinburgh, UK; 4 ICG Software, Pol. Industrial Torrefarrera C. Mestral, , s/n 25123 Torrefarrera, Lleida, Spain

**Keywords:** Cloud systems, e-Health, Queue systems, Quality of service

## Abstract

**Background:**

Cloud computing is a new paradigm that is changing how enterprises, institutions and people understand, perceive and use current software systems. With this paradigm, the organizations have no need to maintain their own servers, nor host their own software. Instead, everything is moved to the cloud and provided on demand, saving energy, physical space and technical staff. Cloud-based system architectures provide many advantages in terms of scalability, maintainability and massive data processing.

**Methods:**

We present the design of an e-health cloud system, modelled by an M/M/m queue with QoS capabilities, i.e. maximum waiting time of requests.

**Results:**

Detailed results for the model formed by a Jackson network of two M/M/m queues from the queueing theory perspective are presented. These results show a significant performance improvement when the number of servers increases.

**Conclusions:**

Platform scalability becomes a critical issue since we aim to provide the system with high Quality of Service (QoS). In this paper we define an architecture capable of adapting itself to different diseases and growing numbers of patients. This platform could be applied to the medical field to greatly enhance the results of those therapies that have an important psychological component, such as addictions and chronic diseases.

## Background

A recent study [1] showed as personalized follow-up by using of telematic tracking applications by means of SMS messaging improved the results in the quitting smokers patients. Related experiments also proved that the same method is useful for application related with the treatment of hypertensive patients [2] and in patients with chronic diseases in general [3]. By using telematic applications, the time dedicated to personalized clinical attention to patients increase, and clinicians more effectively scheduled and managed that time. Also avoids unnecessary travel by patients, while allowing them to feel closely followed by the clinician. This is just one example of the benefits that can bring telematic applications, whose implementation in health centres is increasing.

This article presents the design of a cloud platform with QoS guarantees (based on waiting time for services) applied to e-Health. It is thought to include a wide range of telematic as well as usual programs (administration, specialised, general purpose, etc.). Cloud computing can offer many opportunities to improve health care services from the viewpoint of management, technology, security and legality [4]. By moving the infrastructure to the cloud, valuable data extracted from the different databases of treatment, patients, diseases, and so on will be accessible to doctors to perform analytical studies and see statistical results. By hiding personal patient details, data could be shared between doctors and even hospitals, and could also be cross-reference information from different diseases and treatments. In [5], the authors examine how the biomedical informatics community, especially consortia that share data and applications, can take advantage of cloud computing. Cloud computing systems offer the illusion of infinite computing resources available on demand, allowing an expansion of the resources when needed. Hardware and software services are more efficiently handled than in other High Performance Computing (HPC) infrastructure as they can be added and released dynamically [6]. However, problems arise when scaling the system, this is, when trying to deploy a platform to support the computing needs of many hospitals, with different clinical departments, with their corresponding clinicians and patients. We can say that this health approach can be extrapolated to many other areas, administration, education, social care, etc.

Cloud computing has gained worldwide attention from many researchers, but only a small portion of them have addressed the QoS performance problem [7]. QoS performance includes indicators such as response time, task blocking probability, probability of immediate service, and mean number of tasks in the system [8], all of which may be determined using the tools of queuing theory [9].

We use Cloud computing and queuing system theory to address the problem of cloud scaling. By modelling a queue system we aim to provide scalability to the cloud infrastructure running on a given virtualized platform. Thus the cloud system can automatically scale out in an optimal way in order to guarantee the QoS (e.g. waiting time), planning the proper deployment and removal of virtual machines according to the system load [10]. Platforms like Xen [11] or VMWare [12] offer virtual computing environments that allow for flexible cloud system management and configuration. Despite this, they do not offer tools to manage the computational resources (mainly virtual servers) in a dynamic and flexible way given a defined Quality of Service (QoS). In order to achieve that, OpenStack [13] can be used, an open source software for managing virtual machines.

Quite different, our work does not focus on the investigation of specific queuing theory challenges but on the use of existing models for designing and testing performance of cloud systems in e-Health. We are interested in modelling QoS performance by scaling e-Health cloud platforms, leaving aside other issues such as reliability, security or availability.

### Preliminary concepts and related work

**Figure 1:**
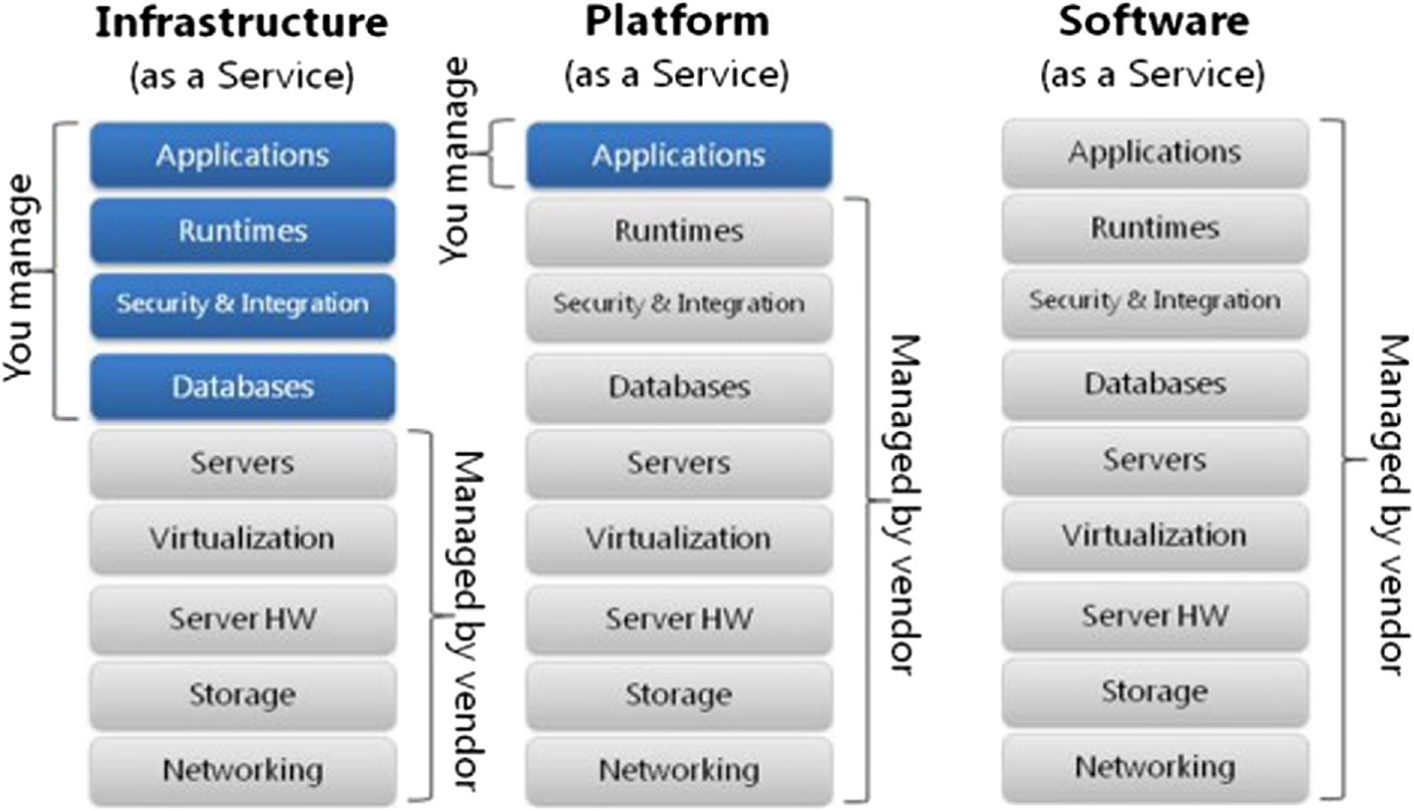
**Cloud services.** Classification of cloud systems according to the services they offer. **SaaS** allows users to run online applications. The vendors own the applications and the users pay a fixed subscription fees. **PaaS** allows users to create their own cloud applications, providing all the execution and compilation of software as well as operating systems. **IaaS** allows users to run any applications they want to on cloud hardware of their choice.

A cloud system is a network of computer servers that are offered under demand as a service, and they are designed to be scalable and flexible. Cloud systems can be served in three different ways (see Figure 1). The first layer is Infrastructure as a Service (IaaS), which means offering hardware, storage and physical devices over the Internet; The second layer is Software as a Service (SaaS), which means offering software and hosted applications over the Internet; And as a combination of both, Platform as a Service (PaaS), which means offering the capability to deploy applications created using programming languages, libraries, services, and tools supported by the provider. The consumer does not manage or control the underlying cloud infrastructure, but has control over the deployed applications [7,14]. In our case, we are interested in modelling a private cloud system, maintained by one organization/institution, of the SaaS kind, which mainly provides software services to its members or end users, clinicians and patients.

In [15], the authors obtained the response time distribution of a cloud system modelled by means of queuing theory on a classical *M/M/m* open network with *m* servers, assuming an exponential density function for the inter-arrival and service times (*M*). By using the response time distribution, they determined the level of service and the relationship between the maximum number of tasks and the minimum number of resources (virtual machines). The response time takes into account both waiting time in the queue and service time. In [16], the authors obtained the response time distribution for a cloud with a *M/M/m/m+r* system model. Having in addition a finite number of buffers (i.e. connections) of size *m+r*. M/M/m/m+r models can be more suitable when we have a known finite buffer for arrivals. M/M/m models are useful when these maximum connections are unknown or not relevant, and the resulting analysis is not as complex as in the M/M/m/m+r models.

The study of the case where the time between arrivals and/or service time does not follow an exponential distribution is much more complex, as for example G/M/m, M/G/m and G/G/m models. Many theoretical studies have been based on extensive research in performance evaluation, including those that analysed the M/G/m model (e.g. [17]). The complexity in these cases comes from the impossibility of obtaining a closed formula to represent the probability distributions of the response or waiting time of customers in the queue, and therefore requires finding approximate models.

As stated in [18], the majority of current cloud computing infrastructure as of 2009 consists of services that are offered up and delivered through a service centre such as a data centre that can be accessed from a web browser anywhere in the world. Our proposal also relies on that.

In this paper, we study a queuing performance model consisting of a cloud architecture (or simply called a cloud) and a service centre such as a data centre. The cloud, is a single point of access for the computing needs of the customers being serviced [18] through a Web browser supported by a Web server. In [15] the service centre was modelled as a collection of service resources used by a service provider to host service applications for customers. In our case, the service centre is a database server. The service provider is required to execute service requests from a customer within negotiated quality of service (QoS) requirements for a given price determined by the service level agreement (SLA). The SLA is a contract negotiated and agreed between a customer and a service provider. In our case the customers will be the end users (clinicians and patients) and the service provider the owner organization of the cloud.

However, traditional queuing results are not directly applicable to performance analysis of cloud computing when one or more of the three following issues holds [7], the number of servers is huge, this is cloud systems made up by hundreds or thousands of nodes [19]; the distribution of service times is unknown, and does not follow a “well-behaved” probability distributions such as exponential distribution; finally, the traffic intensity can vary in an extremely wide range. Cloud centres must provide expected QoS at widely varying loads due to its dynamic nature [15,20], so load peaks are badly modelled by queuing systems.

### Cloud architecture

**Figure 2:**
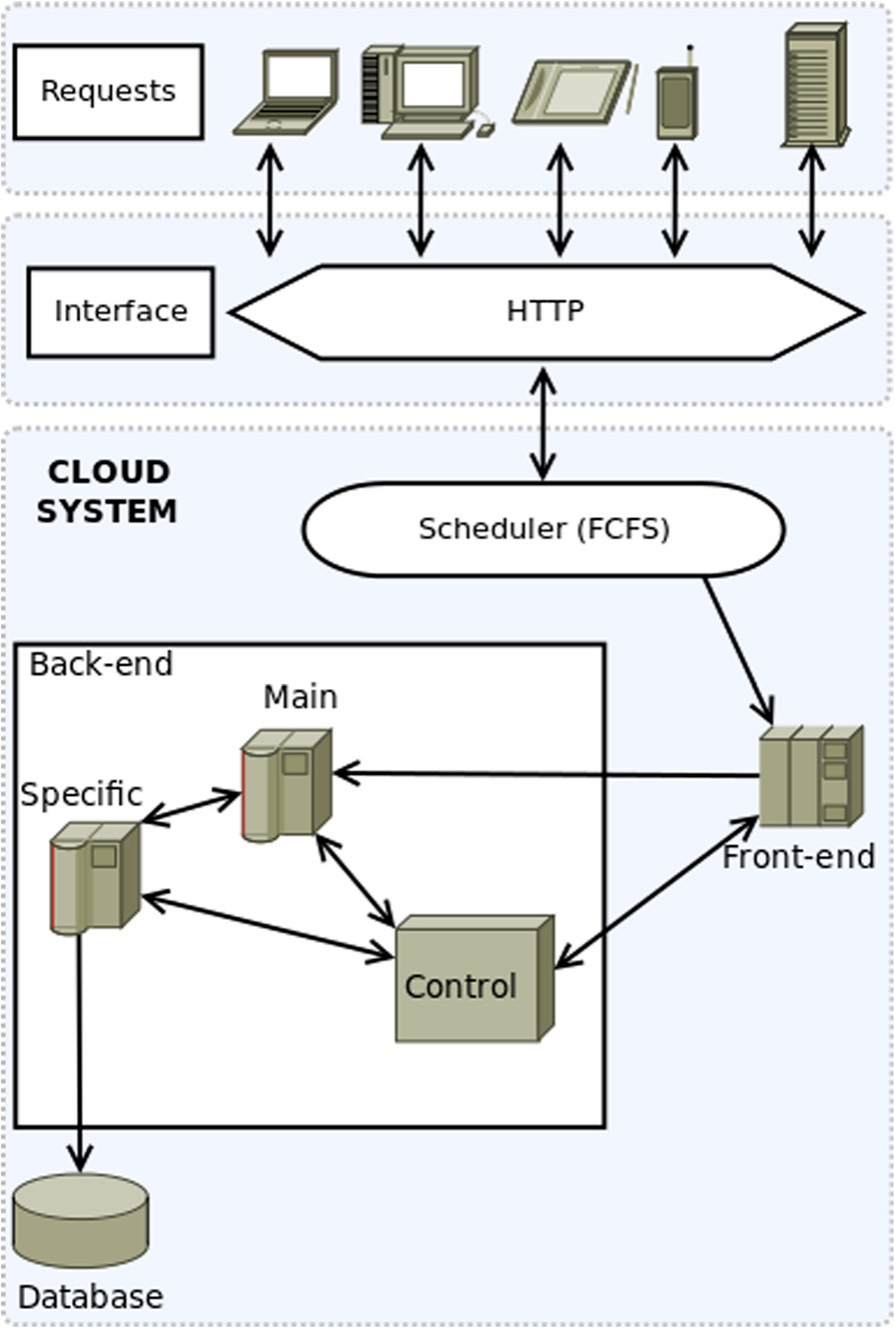
**Cloud system modelling.** Design of the proposed cloud architecture. User requests from multiple devices go through a HTTP interface to the cloud system. A First-Come-First-Serve scheduler distributes all these requests to the Front-end nodes, which forward these to the Back-end nodes. The Back-end nodes process the requests and compute the expected user result, accessing the system database if needed. In the Back-end, there are also control nodes that monitor the state of the system, and are able to create or destroy virtual machines according to that state.

The architecture of our cloud platform consists of two main parts: Front-end and Back-end (see Figure 2).

#### Front-end

The Front-end is the gateway to the cloud and consists of the software components and the interfaces needed to connect to the platform by using remote client applications. These applications usually use standard Web protocols to access the system and an authentication protocol which allows access to authorised users (clinicians and patients). All requests are processed by the scheduler, which sends the selected tasks to the queue of the Back-end. For simplicity, a First Come First Serve (FCFS) scheduling policy was assumed.

As we are proposing a generic system, medical workflows will not be implemented as part of our model. Instead, these medical workflows will be implemented via software. All arriving tasks in our model will consist of web requests, avoiding deadlock situations that could otherwise arise when using a FCFS queue policy.

#### Back-end

The Back-end functions include management of the job queue, the servers and their virtual machines and the storage servers with their database system. Database inconsistencies are avoided by considering only one storage (i.e. database) server. All requests from the Front-end are managed by a scheduler to be allocated in a queue. The server system consists of multiple virtual machines managed by OpenStack and connected to a database server.

The Back-end is made up of three different kinds of servers: **Primary servers:** virtual machines running the multithreading application. The parallel degree of the applications will depend on the threads (tasks making up the application when executed) it can be decomposed. These servers are responsible for performing most of the computation. **Specific Servers:** virtual machines whose main task is to perform specific calculations and handle the Front-end interface. Moreover, they manage the communication with the database and with other servers (even the primary servers). **Control Server:** virtual machine in charge of monitoring the overall system status. This server is responsible for creating and removing virtual machines dynamically.

### OpenStack

The cloud architecture presented in previous section can be implemented with OpenStack [13]. OpenStack is an open source software that provides a massively scalable and pluggable framework for building private and public clouds. Notice that our cloud was characterised as private and scalable, so it ideal for our purpose. It goes beyond a classic hypervisor (i.e. VirtualBox [21], Xen [11], VMware [12]), and allows the setup of virtual machines dynamically, as computational resources are needed. This guarantees high QoS in periodic traffic spikes, when the arrival rate of the requests to be served increases. OpenStack can be set up to create new instances when current servers are overwhelmed and to shut them down when traffic decreases. This feature ensures you that the number of instances in the cloud system scales up when your system grows, and is particularly well suited for applications that experience deep variability in usage.

OpenStack offers a set of APIs (Application Programming Interface) that allow to interact dynamically with the installed OpenStack platform. Using these APIs, it is possible to authenticate and interact with the system from the command line or programmatically. For example, in Python we have available the python-nova client API [22,23] avaialble, where the *nova boot* and *nova delete* commands allow us respectively to boot a new server and immediately shut down and delete a server dynamically.

## Methods

### System analysis and design

The main aim of this work is the design of the Back-end, composed of the primary, specific and control servers. The design has to take into account the analysis of requirements, which in our case exclusively focus on the characterisation of arrival frequency of the users and the QoS in serving them with our cloud platform.

The e-Health application we are targeting must be scalable in order to provide a service to an unlimited number of users which will be mainly healthcare staff and patients from various hospitals. Taking into account the cloud architecture (Section Back-end), the *primary* servers of the Back-end are the ones in charge of serving the platform users’ requests.

Furthermore, several *specific servers* will be in charge of the communications with the database containing the healthcare information.

Finally, the *control server* will be in charge of managing the creation and disposal of the *specific* and *primary* servers. In order to control the system we propose the creation of a queuing system that models system performance. This model is described in Section *Modelling*.

Figure 2 shows the design of the cloud system, including how service requests are planned by the “Scheduler” via a FCFS queue. Then, the requests are forwarded to the Front-end in charge of submitting tasks to the Back-end. Finally, the communication among the Back-end components is also shown.

### Modelling

In this section, we will focus only on the Back-end, which is managed by the *control server*. Its basic function is to create and remove *specific* and *primary* servers. These decisions are taken according to the waiting time of the user tasks.

**Figure 3:**
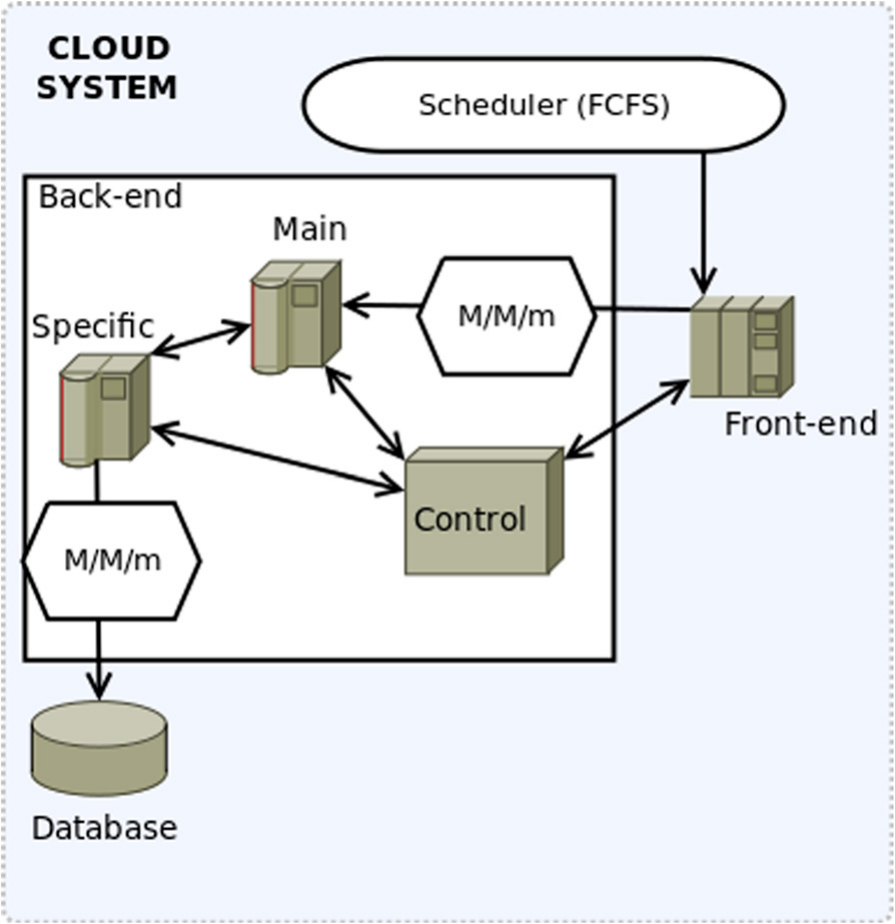
**Model.** Graphical representation of the two system queues. Both of them are of the same type (*M/M/m*). The first *M/M/m* queue models the access to the primary servers, and is always accessed when new requests enter the system. The second *M/M/m* queue models the access to the database cluster, which is accessed based on a probability depending on the Back-end nodes.

As can be seen in Figure 3, the system will contain two queues of the same type (*M/M/m*). This means that both the time between user arrivals to the system and the service time of the system follow an exponential distribution with means *λ* and *μ* respectively, with *m* servers with an FCFS scheduling policy. The first queue models the *primary servers* while the second one models the *specific servers* that interact with the database.

**Figure 4:**
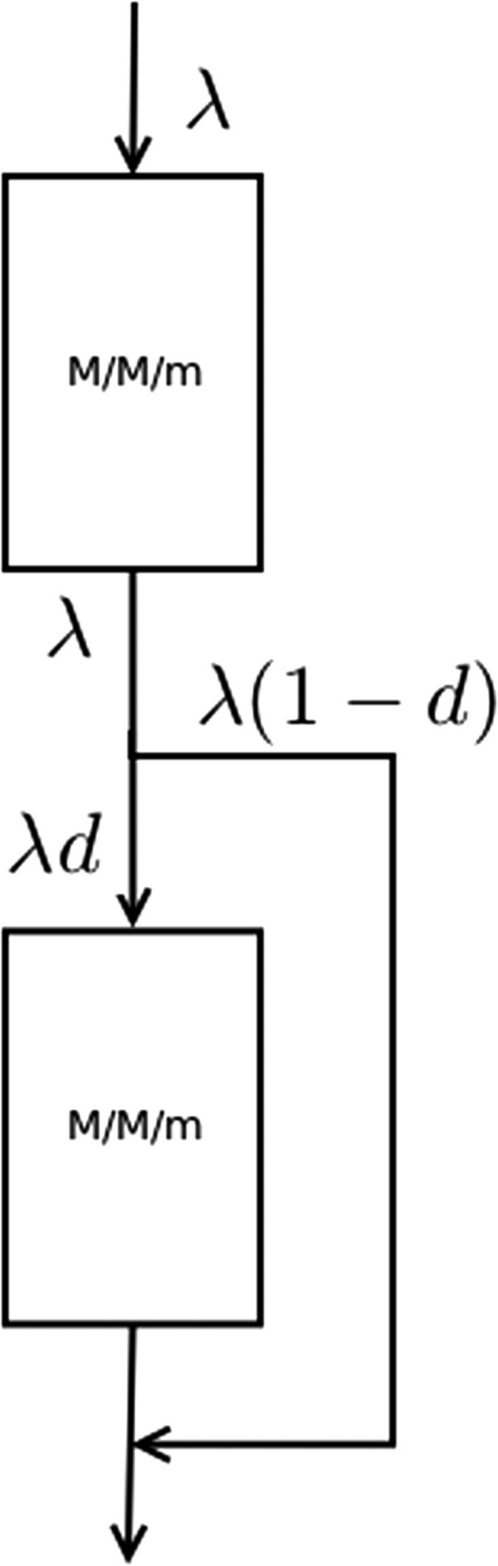
**Two serially connected*M/M/m* queues.** Queueing system composed by two *M/M/m* queues connected serially. The first one models the access to the primary servers, and the second one models the access to the database. *λ* is the request arrival rate. There is a probability *d* of accessing the second queue, and a probability (1−*d*) of exiting the queueing system without going through the second queue.

Abstracting away the details of the application problem, we propose a model of a queueing system composed by two *M/M/m* queues connected serially, as can be seen in Figure 4. The user tasks enter the system though the first queue; then they move on to the second queue (this represents the database system) with probability *d*. Conversely, a user has (1−*d*) probability of leaving the system without passing through the second queue. In this way, we are modelling a system in which each user requires a computing operation and a database access with probability *d*.

According to Burke’s theorem [24], the output of a stable *M/M/m* queue with an input parameter *λ* and a service parameter *μ* for each one of the *m* servers is a Poisson process with the same input parameter *λ*. This means that the serial connection of two *M/M/m* systems (without cycles) is independent between them and these systems keep the same density distributions, both for arrival and service.

Our two queues can be analysed independently, and they form an open Jackson network. The interconnection and behaviour between the queues is ruled by Burke’s [25] and Jackson’s theorems. Burke states that we may connect many multiple-server nodes together in a feedforward network and still preserve the node-by-node decomposition. Jackson [26,27] states that to calculate the total average arrival rate we must sum the arrivals from outside the system plus arrivals from all internal nodes.

#### M/M/m

**Figure 5:**
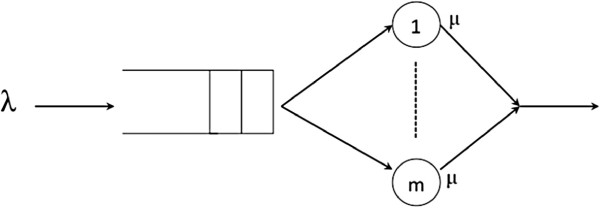
***M/M/m* queue scheme.** Representation of an *M/M/m* queuing system with *m* servers and two density functions. The average arrival rate of the requests is represented by *λ*. The total number of servers goes from one to *m*, each one having a service rate represented by *μ*.

In this section we analyze the *M/M/m* queuing system, with *m* servers and two density functions, that represents the average arrival (*λ*) and service rate per server (*μ*), as can be seen in Figure 5.

**Figure 6:**
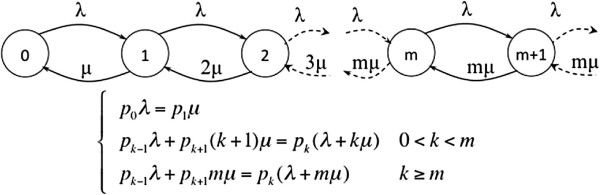
**Transition state diagram and equilibrium equations of*M/M/m* queue.** State transition diagram of the *M/M/m* queue and the equilibrium equations that define it. The state space records the number of customers in the queueing system. The values *λ* and *μ* represent the arrival and service rates of customers.

Figure 6 shows the state transition diagram of the system in equilibrium, as well as the equations that define it.

Solving the system of equations we can obtain the value for *p*_*k*_, i.e., the probability of the system having exactly *k* users.

(1) $$p_{k}=\left\{\begin{array}{ll} p_{0}\frac{{\left(\text{mp}\right)}^{k}}{k!} & \;k\leq m\\ p_{0}\frac{m^{m}\rho^{k}}{m!} & \;k\geq m \end{array}\right)$$

where the utilisation factor (*ρ*) is:

(2) $$\rho =\frac{\lambda }{\mathrm{m\mu}}<1$$

Taking into account that:

(3) $$\overset{\infty }{\underset{k=0}{\sum }}p_{k}=1,$$

we obtain the probability of having no users in the system (*p*_0_):

(4) $$p_{0}={\left[\sum_{k=0}^{m-1}\frac{{\left(\mathrm{m\rho}\right)}^{k}}{k!}+\frac{{\left(\mathrm{m\rho}\right)}^{m}}{m!(1-\rho )}\right]}^{-1}$$

The average number of users in the waiting queue (*N*_*W*_) is:

(5) $$\begin{array}{ccc} N_{W} & = & \overset{\infty }{\underset{k=0}{\sum }}kp_{k+m}\\ & = & \overset{\infty }{\underset{k=0}{\sum }}kp_{0}\frac{m^{m}\rho^{k+m}}{m!}=\frac{p_{0}{\left(\mathrm{m\rho}\right)}^{m}}{m!}\overset{\infty }{\underset{k=0}{\sum }}k\rho^{k}\\ & = & \frac{p_{0}{\left(\mathrm{m\rho}\right)}^{m}}{m!}\frac{\rho }{{\left(1-\rho \right)}^{2}} \end{array}$$

The average waiting time in the queue *W* (this is the QoS parameter we have chosen for this work) is defined as:

(6) $$W=\frac{N_{W}}{\lambda }$$

#### Quality of service

As was said before, the selected Quality of Service (QoS) criterion is the waiting time in the queue. This waiting time depends on the utilization factor *ρ*. In an *M/M/m* system queue, $\rho =\frac{\lambda }{\mathrm{m\mu}}$.

According to the guidelines stated by Shneiderman [28-30], a system’s response time should be appropriate to the task that is being performed. For typing, cursor motion and mouse selection, they define an interval of between 50 and 150 milliseconds, and a value of 750 to 1000 milliseconds for simple and frequent tasks. The customers of our system will be performing simple and frequent tasks due to the interaction with a web-based application. For these reasons, we assume a *W*_*min*_ value of 150ms and a *W*_*max*_ value of 750ms. These values could be modified to analyse other kinds of system where mean acceptable response times could be different.

As a consequence, we can establish that if the average waiting time of our system is longer than *W*_*max*_, the system will have to create new virtual machines, this is, to increase the number of *primary* servers or, depending on the case, *specific* servers until *W* returns back under the *W*_*max*_ threshold. Conversely, if *W* drops below the *W*_*min*_ value, the system can release resources, which in our case corresponds to removing *primary* (or *specific*) *servers*, until *W* is again above the lower limit *W*_*min*_. This mechanism is implemented in the algorithm presented in Quality of service section which checks every period of time *T* the value of *W* and determines the need for creating or removing *primary* or *specific* servers until *W* lies within the range *W*_*min*_ ≤ *W* ≤ *W*_*max*_ range. Currently, *T* is a predetermined value, set by the system administrator, but it would be interesting to calculate *T* in function of *ρ*. In the same way, it would also be interesting to incorporate some kind of control mechanism in the algorithm in order to decide which type of virtual machines (primary or specific) should be created or removed when necessary.

## 

### Algorithm 1 QoS control

## Results and discussion

**Figure 7:**
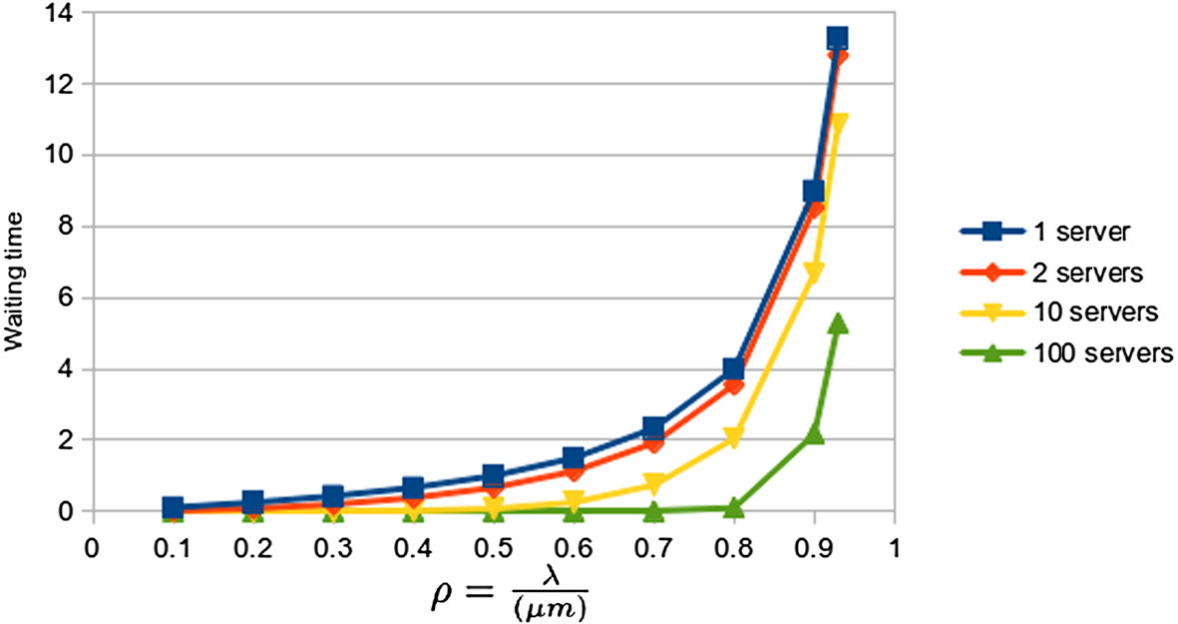
**Waiting time on queue 1 (M/M/m).** Graph plotting how the waiting times (in generic units) of the first queue increases by increasing the occupation ratio *ρ* for one, two, ten and a hundred servers. It shows that increasing the number of servers significantly decreases the resulting waiting time.

The following section presents an analysis about how the response time is affected by increasing the number of servers in an *M/M/m* queue. Figure 7 shows how the waiting times (in generic units) of the first queue increases by increasing the occupation ratio *ρ* for one, two, ten and a hundred servers. These values have been obtained by using the queue simulator server Queue 2.0 [31].

**Figure 8:**
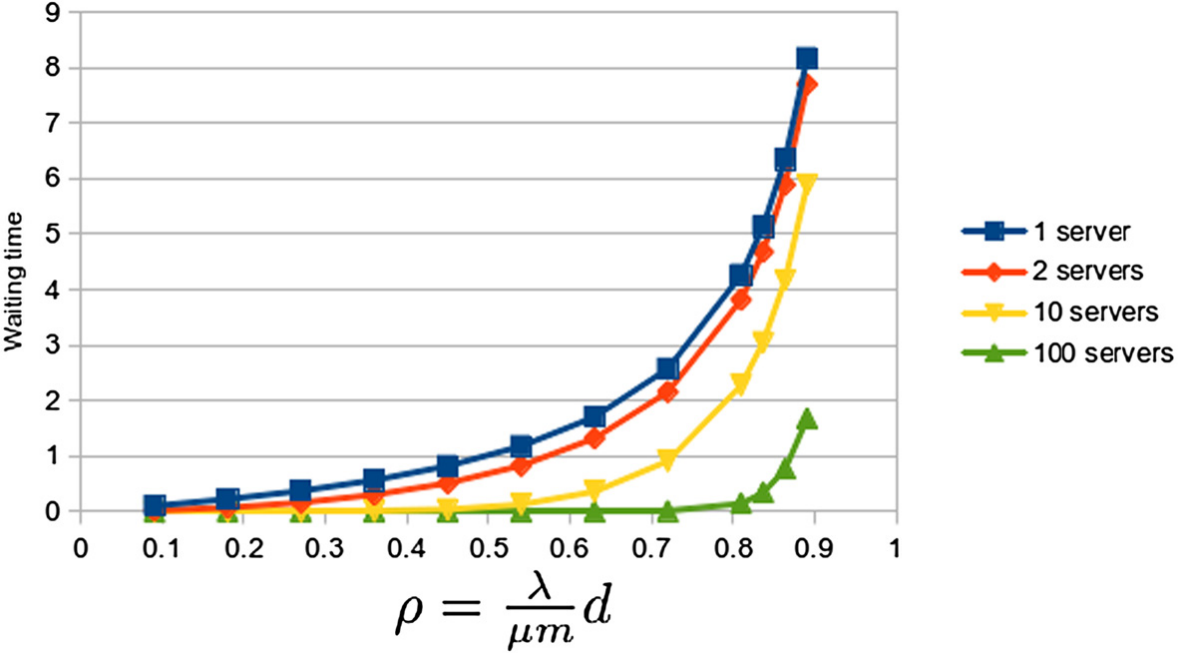
**Waiting time on queue 2 (M/M/m).** Graph plotting how the waiting times (in generic units) of the second queue increases by increasing the occupation ratio *ρ* for one, two, ten and a hundred servers. It shows that increasing the number of servers significantly decreases the resulting waiting time.

For the second queue, the entering rate is based on *λd*. Figure 8 shows the same results as Figure 7 by using instead the second queue. We have assumed a value of *d* = 0.9 as the probability of one user accessing the database servers.

The mean access rate to the database *d* can widely vary from one application to another. We have assumed a 0.9 value due to our experimental application making requests to the database for 90% of the user requests. We also did some testing with slightly modified values of *d* and proportional results were obtained.

As was expected, the waiting time of the queue 2 is smaller than that of queue 1. As the user flow lowers in the queue 2 also decreases its mean waiting time. Thus, waiting times decreases with *d*. *W* is the sum of waiting times in queue 1 and 2. *W*_*max*_ and *W*_*min*_ will determine the number of clients/connections to be served simultaneously. For example, we could set *W*_*max*_ = 13 if this generic time value corresponds to 750ms in the real cloud. It has been shown how a widely used and tested kind of queuing model can be used to model cloud computing systems.

**Figure 9:**
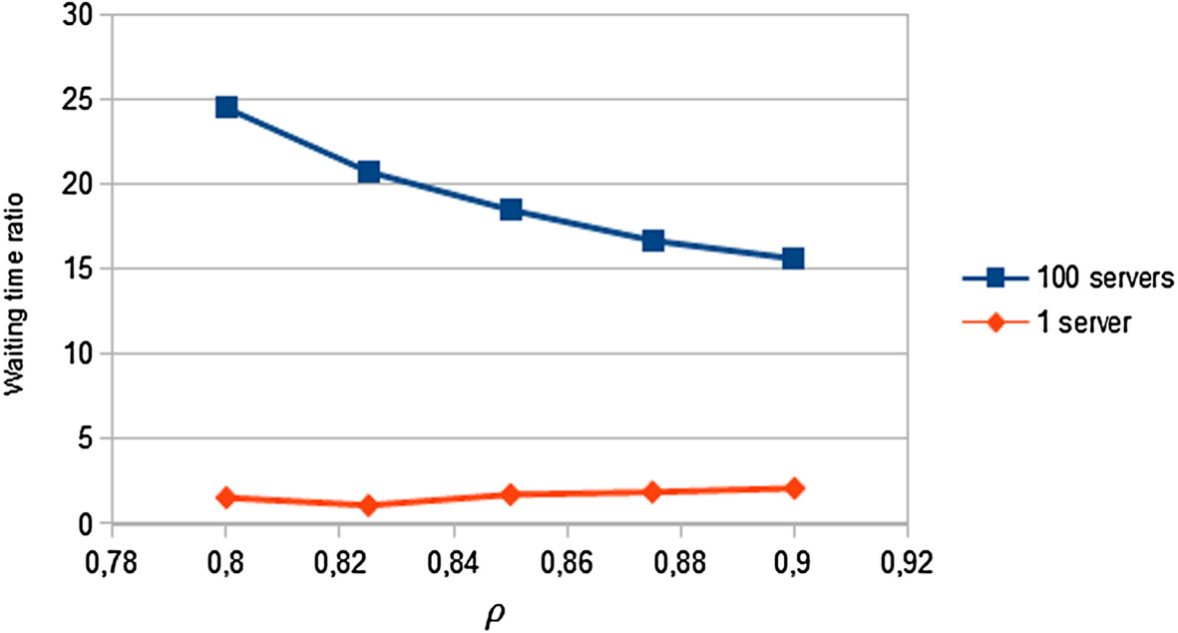
**Waiting time rate.** Graph plotting the waiting time ratio between waiting times of the first and second queues with one and a hundred servers, for different *ρ* values.

We would like to highlight that for small numbers of servers, the relation between the waiting time of both queues does not change, keeping it at constant levels. On the other hand, for large computing systems, with huge computing requirements (virtual servers), the waiting time between the first and the second phase tends to stabilize when we increase the parameter *ρ*. Furthermore, as Figure 9 shows, this relation suffers a significant drop in large systems with high number of virtual machines. This explain why queuing systems cannot be applied to model huge cloud farms of servers.

## Conclusions

In this paper, a new application of cloud computing paradigm is presented by designing a system model applied to e-Health. The design of a cloud system requires the use of scalable, centralized, flexible, and dynamic architectures with a high level of integration. We have selected queuing theory as the basic mean to model the performance of the cloud system. As a result, the dynamics of the system based on the creation/deletion of the virtual systems is controlled by a queuing system model.

Through a preliminary analysis, the design of a cloud architecture with e-Health requirements has been proposed. The combination of two systems M/M/m in sequence has been proposed to model the cloud e-Health platform. The first offers compute services, and the second provides service access to a database server. Our work has shown that to provide good QoS, in terms of averaged waiting times, the the waiting time between the first and the second phase tends to stabilize. This reduction becomes much more significant when we increase the number of virtual machines.

The proposed system can improve the e-Health field by providing a model to support medical software, saving resources and enhancing the control and management of the patients. Pay-per-use service would lower overall costs. The proposed system would also allow tendencies that could be used to improve the current treatments to be generated and analysed. Also, having an electronic clinical history would save paper, physical space and would improve the efficiency of those who need to access it. The design can easily satisfy the needs of e-Health related applications without major changes, allowing the construction of web-based applications that implement all the needed medical workflows. The proposed system guarantees high scalability, ensuring that when the system requirements grow, the underlying platform will be able to handle the situation. Also, the proposed system suggests the usage of a large shared infrastructure, which would result in many hospitals and treatments having homogeneous data that would facilitate correlations and data mining.

### Future work

As explained above, we would like to extend the algorithm presented in Quality of service section to determine the value of *T* based on *ρ*. We would like to run more tests in order to explain how fast can W (waiting time) change and the proposed system reaction to these changes. Furthermore, it would be of great interest to incorporate mechanisms for deciding the type of virtual machines that should be created/deleted (primary or specific servers). Moreover, we would like to replace both queues with a more realistic *M*/*M*/*m*/*m* + *r*/*K* model, with *m* servers, *m* + *r*user connections (the maximum number of users in the system, that is, users receiving the service, being at most *m*, plus users who are waiting, at most *r*), and a maximum number of *K* users as presented in [7]. In our case, if patients can enter the system, a *M/M/m* system could be used, as we would have not a clear reference to the maximum number of users in the system. In the other case, if patients can not enter the system, we could take the *M*/*M*/*m* + *r*/*K*approach because we would have a more specific set of customers. We would want to create an adaptive system that could select the best model for each situation. As future work, we also plan to develop an application by using OpenStack, which will emulate the requirements of the Tobacco Control Unit in Santa Maria Hospital (Lleida, Spain), using real data based on user numbers and requirements. We have already implemented a preliminary prototype [32]. The aim of this work would be to estimate the computing resources that such a Tobacco Control Unit would require. In this way, by knowing the hospital users, we will design a cloud system applied to e-Health in a specific hospital. This application should be extended to emulate the behaviour of the system assuming the scalability of the system by increasing the number of hospitals. We would also like to extend the scalability tests to more than one hundred servers. We would like to test up to one million servers in order to verify the scalability of the system.

## Competing interests

The authors declare that they have no competing interests.

## Authors’ contributions

JV, RF and DR contributed to the study concept and design of the experimental tests. JV and JR performed the experimental tests and the data analysis. FS contributed to the development of the model and the algorithms presented in this paper, and took the lead in drafting this paper. FS, JV and JR wrote the first version of the manuscript. All five authors contributed to the preparation of the manuscript. All authors read and approved the final manuscript.

## Authors’ information

JV received his BS and MS in computer science from Universitat de Lleida (UdL) in 2006 and 2008 respectively. Currently he is a PhD student in the same University and his research interests are Cloud computing, e-Health, and parallel simulation.

FS received the B.S., M.S. and Ph.D. degrees in computer science from the Autonomous University of Barcelona, Spain, in 1991, 1994 and 2002 respectively. At the present time, he is an associate professor in the Department of Computer Science at the University of Lleida (Spain). His research interests include distributed processing and HPC.

JR received his B.S., M.S. and Ph.D. in computer science from University of Lleida (UdL) in 2006, 2008 and 2012 respectively. Currently he is leading the research division at ICG Software and he is an assistant lecturer at University of Lleida. His main research interests are high-performance computing, P2P systems and Cloud computing.

RF received the MS degree in computer science from the University Deusto of Bilbao in 2003 and the Ph.D degree in computer science University Carlos III of Madrid in 2010. She had been an assistant professor since 2004 at the Universidad Carlos III de Madrid. Nowadays she is working as Research Assistant in University of Edinburgh. Her main reseach interest are high performance computing, data stream transfer and Cloud Computing.

DR received his BS and PhD in Physics from University of Cantabria in 1998 and 2007 respectively. Currently he is an associate researcher at the Edinburgh Data Intensive Research group (School of Informatics) and the Brain Research Imaging Centre (Division of Clinical Neurosciences) of the University of Edinburgh. His main research interests are information governance, privacy protection in the e-Health context and data intensive science.

## Pre-publication history

The pre-publication history for this paper can be accessed here:

http://www.biomedcentral.com/1472-6947/13/35/prepub
